# Prognostic potential of lipid profiling in cancer patients: a systematic review of mass spectrometry-based studies

**DOI:** 10.1186/s12944-024-02121-0

**Published:** 2024-05-25

**Authors:** Yusuke Takanashi, Tomoaki Kahyo, Keigo Sekihara, Akikazu Kawase, Mitsutoshi Setou, Kazuhito Funai

**Affiliations:** 1 First Department of Surgery, Hamamatsu University School of Medicine, 1-20-1 Handayama, Chuo- ku, Hamamatsu, Shizuoka 431-3192 Japan; 2 Department of Cellular and Molecular Anatomy, Hamamatsu University School of Medicine, 1-20-1 Handayama, Higashi Ward, Hamamatsu, Shizuoka 431-3192 Japan; 3 International Mass Imaging Center, Hamamatsu University School of Medicine, 1-20-1 Handayama, Chuo-ku, Hamamatsu, Shizuoka 431-3192 Japan; 4 Department of Systems Molecular Anatomy, Institute for Medical Photonics Research, Hamamatsu University School of Medicine, 1-20-1 Handayama, Chuo-ku, Hamamatsu, Shizuoka 431-3192 Japan

**Keywords:** Cancer, Prognostic prediction, Lipid, Lipidomics, Mass spectrometry

## Abstract

Cancer prognosis remains a critical clinical challenge. Lipidomic analysis via mass spectrometry (MS) offers the potential for objective prognostic prediction, leveraging the distinct lipid profiles of cancer patient-derived specimens. This review aims to systematically summarize the application of MS-based lipidomic analysis in prognostic prediction for cancer patients. Our systematic review summarized 38 studies from the past decade that attempted prognostic prediction of cancer patients through lipidomics. Commonly analyzed cancers included colorectal, prostate, and breast cancers. Liquid (serum and urine) and tissue samples were equally used, with liquid chromatography–tandem MS being the most common analytical platform. The most frequently evaluated prognostic outcomes were overall survival, stage, and recurrence. Thirty-eight lipid markers (including phosphatidylcholine, ceramide, triglyceride, lysophosphatidylcholine, sphingomyelin, phosphatidylethanolamine, diacylglycerol, phosphatidic acid, phosphatidylserine, lysophosphatidylethanolamine, lysophosphatidic acid, dihydroceramide, prostaglandin, sphingosine-1-phosphate, phosphatidylinosito, fatty acid, glucosylceramide and lactosylceramide) were identified as prognostic factors, demonstrating potential for clinical application. In conclusion, the potential for developing lipidomics in cancer prognostic prediction was demonstrated. However, the field is still nascent, necessitating future studies for validating and establishing lipid markers as reliable prognostic tools in clinical practice.

**Supplementary Information:**

The online version contains supplementary material available at 10.1186/s12944-024-02121-0.

## Introduction

Dysregulation of lipid metabolism is a metabolic hallmark in cancerous tissues [1, 2]. Lipids play various roles in maintaining characteristics unique to cancerous tissues, and their consumption and supply are elevated in these tissues [3, 4]. To support the high proliferative capacity of cancer cells, the expression of choline kinase, an enzyme involved in the synthesis of phosphatidylcholine (PC) and phosphatidylethanolamine (PE), which are major lipids constituting the cell membrane, is found to be upregulated in various types of cancer [5–8]. Mediators like sphingosine-1-phosphate (S1P), phosphatidylinositol (PI), and prostaglandin (PG) E2, which respectively activate signaling pathways such as signal transducer and activator of transcription 3 (STAT3), phosphoinositide 3-kinase/protein kinase B (PI3K/AKT) pathway, and RAS pathway, are also upregulated in cancer tissues, facilitating cancer cell proliferation [9–13]. Enhanced de novo synthesis of fatty acids (FAs) in cancer cells increases the content of saturated and monounsaturated FAs in cell membranes, contributing to oxidative stress resistance [14]. Under stress conditions like hypoxia, cancer cells form intracellular lipid droplets, storing FAs, triglycerides (TGs), and cholesterol to acquire resistance to energy depletion [3, 4]. The formation of these droplets is known as a phenotype of highly malignant cancers. Thus, a distinct lipid profile in cancer tissues reflects biological properties such as malignancy [15, 16].

Clinical judgement including treatment strategies depends on the prognostic predictions which consist with tumor malignancy and progression. However, existing prognostic predictors, such as pathological findings and the TNM classification, rely on subjective judgments and lack reproducibility [17–19]. Therefore, establishing novel, objective prognostic predictors to assist clinical judgment is necessary. Against this backdrop, recent attempts have been made to apply lipidomics—analyzing the characteristic lipid profile of cancer— for cancer prognosis prediction based on insights into lipid metabolic aberrations reflecting the biological characteristics of cancers [20, 21].

Lipidomics aims to analyze lipid classes, including their molecular species, in biological systems comprehensively and quantitatively [22]. As mentioned, because lipids reflect the biological characteristics of cancer, applying lipid profiles as biomarkers can provide additional information not previously available, potentially aiding clinical judgment [21]. The most utilized analytical method in lipidomics has been mass spectrometry (MS), which yields extensive structural information of lipid molecules [22]. With the advancement of MS methods [3], research attempting to apply lipidomics for cancer prognosis prediction have shown a marked increase over the last decade. However, there are no specialized reviews on research applying lipidomics for cancer prognostic prediction, making it difficult to overview the current state, prospects and future subjects of this research field.

This systematic review aims to summarize research from the past decade that attempted prognostic prediction of cancer patients through mass spectrometric analysis of lipids in cancer patient-derived specimens (including annual trends in report numbers, risk of research bias, types of cancers studied, countries where the research was conducted, specimens and mass spectrometric methods used, cohort size and design of the studies, and lipid markers identified as prognostic factors) and to derive future prospects.

## Methods

We conducted study search using PubMed (https://pubmed.ncbi.nlm.nih.gov/) with search-period from August 2013 to September 2023. For searching studies that focus on prognosis prediction of cancer patients by lipid profiling using MS analysis, we employed a following combination of search terms: “lipid” [All Fields] AND “mass spectrometry” [All Fields] AND (“cancer” [All Fields] OR “carcinoma” [All Fields]) AND (“recurrence” [All Fields] OR “prognosis” [All Fields] OR “survival” [All Fields] OR “progression” [All Fields]). Our inclusion criteria were as follows: primary studies assessing on prognosis prediction of cancer patients by lipid profiling; MS was employed as an analytical platform; human-derived specimen, such as cancerous or normal tissue, serum, urine, exosome, was used for lipid analysis; studies published in English. The term ‘prognosis’ encompassed various prognostic aspects including survival, disease/progression-free period, recurrence, stage, TNM classification, and pathological prognostic factors. The exclusion criteria were as follows: evaluated outcomes do not include prognostic information; prognosis prediction models include molecules other than lipids analyzed by MS; studies based on animals or cell-lines; studies written in languages other than English; Review article or meta-analysis. Based on the inclusion and exclusion criteria as mentioned above, we screened the potentially relevant studies by reviewing title and abstract on electronic search. After exclusion of the irrelevant studies, we assessed full-text of the extracted relevant studies for eligibility. The QUADAS-2 tool [23] was used to evaluate the quality and risk of bias of the finally included articles. We extracted following data items from the finally included studies to summarize the study characteristics: cancer type; histological type; year of publication; country where the study was conducted; sample type; analysis platform; molecular identification method; study size; presence or absence of validation using independent cohort; observed lipid biomarkers; associated prognosis or prognostic factors.

## Results

### Study search and annual trends in the number of published studies

**Fig. 1:**
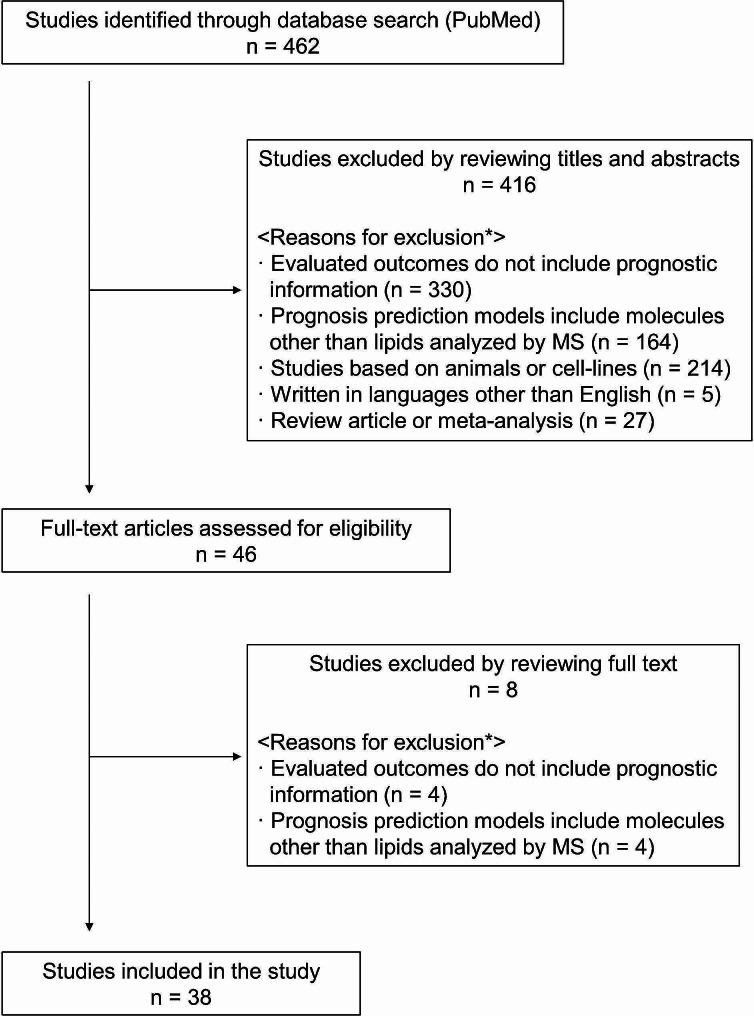
Flow diagram of the study search. Abbreviation: MS, mass spectrometry. *There were studies that meet multiple exclusion criteria

**Fig. 2:**
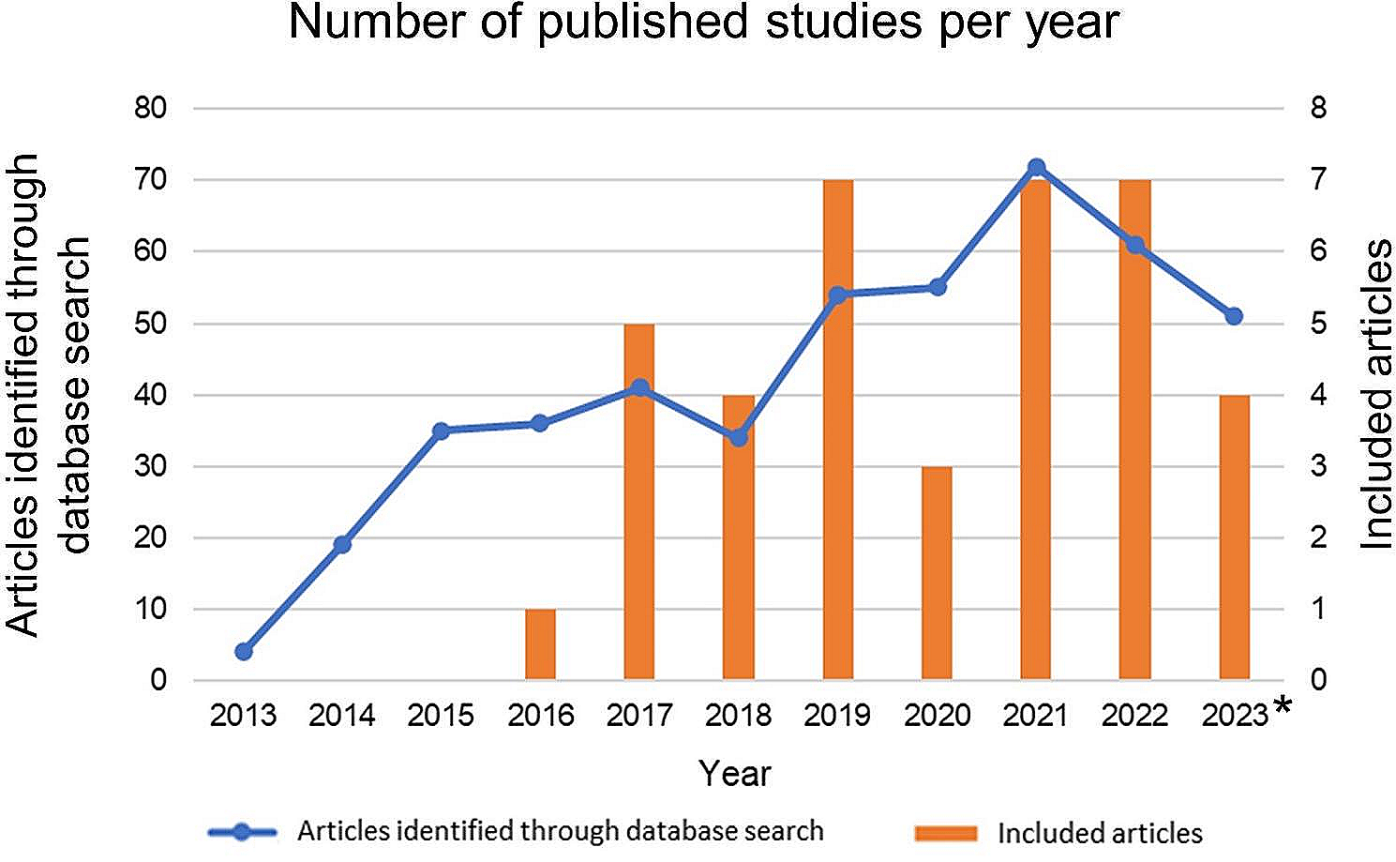
The annual trends in number of studies identified through the database search was on an increasing trend from 2013 (blue line). The annual number of published studies that address prognosis prediction of cancer patients by lipid profiling using mass spectrometry analysis is on an increasing trend from 2016 (orange bar) *The published articles in 2023 are limited from January to September

A flow diagram of the study search is shown in Fig. 1. Our search through PubMed identified 462 potentially relevant studies. Of these, 416 studies were excluded by reviewing the title and abstract. The remaining 46 studies were assessed for eligibility by full-text review, and eight were excluded. Finally, 38 studies that met the inclusion criteria were included in this study. The annual trends in the number of studies identified through the database search have increased since 2013. Also, the annual number of included studies that address prognosis prediction of cancer patients by lipidomics using MS analysis has been increasing since 2016 (Fig. 2).

### QUADAS-2 evaluation

**Fig. 3:**
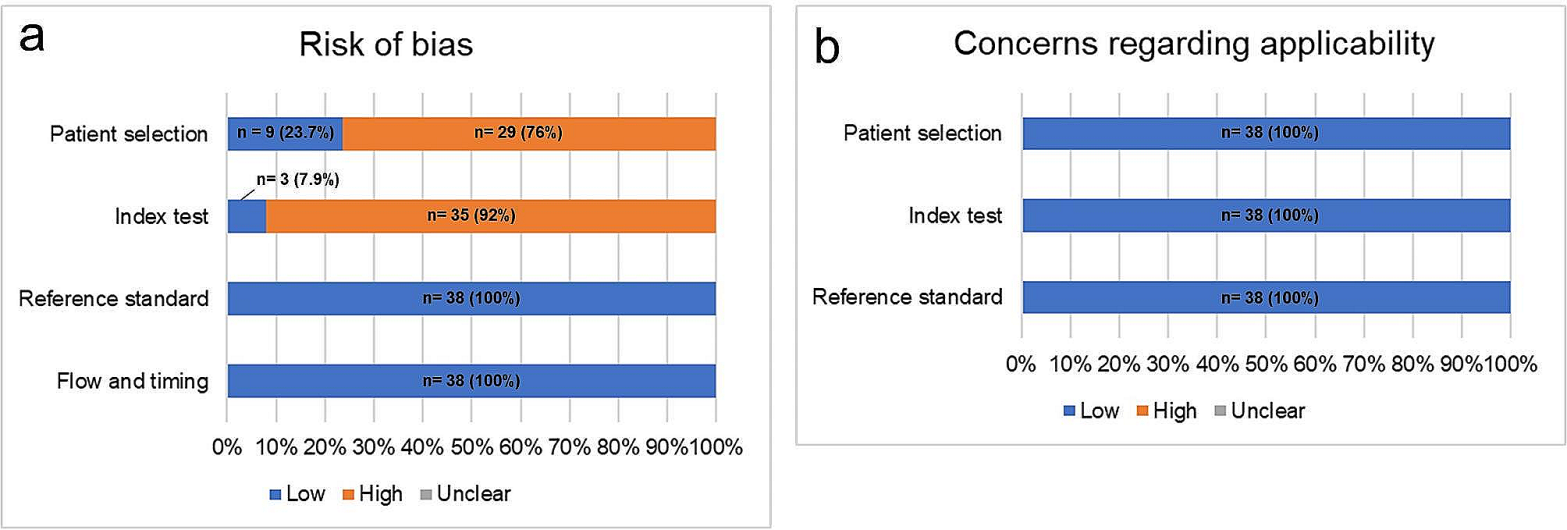
Graph showing the proportion of the included articles with varying risk of bias (**a**) and applicability (**b**) of the QUADAS-2 domains

The results of the QUADAS-2 assessment of the included studies are shown in Fig. 3. In evaluating the risk of bias (Fig. 3a), 76% of the studies were evaluated to have a ‘high’ risk of bias regarding the ‘patient selection’ mainly attributable to retrospective patient selection and adopted case-control design. 92% of the studies had a ‘high’ risk of bias with respect to the ‘index test’: this is due to the necessity of using diagnostic results based on existing diagnostic methods as the reference standard for evaluating the results of the index test, and impossibility to pre-specify the threshold of the index test due to the prevalence of exploratory studies without validation cohorts. All of the studies had a ‘low’ risk of bias regarding ‘reference standard’ and ‘flow and timing’. In evaluating concerns regarding applicability (Fig. 3b), all of the studies had ‘low’ concerns for ‘patient selection’, ‘index test’, and ‘reference standard’ as these matters were addressed with a study design that matched the review question. The detailed evaluation results are presented in the Supplemental file.

### Study characteristics

Study characteristics of the 38 included studies are summarized in Table 1. Below, we describe the results for each extracted study characteristic.

**Table 1:** Summary of characteristics of the 38 included studies

Cancer type	Reference	Country	Sample types	Analysis platform	Molecular identification methods	Study size	Validation cohort	Multi-center cohort	Prognostic outcomes	Observed lipid biomarkers*	
										High	Low
Colorectal cancer	Zheng et al. 2022	China	Tissue	iEESI-MS	Database (NIST Mass Spectral Library)	Tumor *n* = 86	-	-	Advanced stage	-	LPC (18:0)
						Normal tissue *n* = 86					
	Ecker et al. 2021	Germany	Tissue	FIA-MS	Software (ALEX)	Training cohort: tumor *n* = 106 / control *n* = 41	+	+	Shorter disease-free survival Higher lymphatic vessel invasion	TG	
						Validation cohort 1: tumor *n* = 28 / control *n* = 24					
						Validation cohort 2: tumor *n* = 20 / control *n* = 20					
	Sun et al. 2022	China	Serum	LC-MS	Software (Analyst)	Cancer patients *n* = 236	-	-	Shorter overall survival	Cer (d18:0_14:0), Ganglioside GT3 (d18:0_18:1) LPE (22:6_0:0), PS (20:4_14:1)	-
	Rachieriu et al. 2021	Romania	Serum	LC-MS	Database (HMDB, LIPID MAPS)	Cancer patients *n* = 25 / Control *n* = 16	-	-	Advanced stage	Cer (d18:3_20:1), FA (17:2), hexadecanoylcholine PA, PC, PE, stearyl palmitate, SM (d18:1_16:1)	-
	Liu et al. 2019	China	Serum	LC-MS	Software (MS-DIAL)	Cancer patients *n* = 40	-	-	Advanced stage	TG, CE (20:4)	FAHFA (9:0_18:1)
	Figueiredo et al. 2018	Brazil	Serum	MALDI-TOF MS	Database (LIPID MAPS)	Cancer patients *n* = 36 / Control *n* = 37	-	-	Shorter overall survival	-	DG, sphingolipid
Prostate cancer	Ravindran et al. 2022	USA	Tissue	LC-MS	Software (Analyst)	High Gleason grade: tumor *n* = 10	-	-	Higher Gleason grade	Ratio of TG to LPC, SM	-
						Low Gleason grade: tumor *n* = 20					
						Biochemical recurrence: tumor *n* = 5			Higher biochemical recurrence	PI	LPC, PC
						No recurrence: tumor *n* = 10					
	Koizumi et al. 2019	Japan	Tissue	LC-MS	Tandem MS	Tumor *n* = 16	-	-	Advanced stage	PI with 0-2 bonds in acyl chains	-
	Scheinberg et al. 2023	Australia	Serum	LC-MS	Tandem MS	Training cohort: cancer patients *n* = 105	+	+	Shorter overall survival	Cer (d18:1_18:0) Ratio of Cer (d18:1_24:1) to Cer (d18:1_24:0)	total cholesterol, TG
						Validation cohort: cancer patients *n* = 183					
	Clendinen et al. 2019	USA	Serum	LC-MS	Database (HMDB, METLIN) Software (Lipid Search)	Recurrence *n* = 40 / Remission *n* = 40	-	-	Higher biochemical recurrence	AcCa, Cer, DG, LPA, LPC, LPE, LPI, LPS PC, PE, PET, PG, PI, SM, SoP, TG	-
	Lin et al. 2017	Australia	Serum	LC-MS	Tandem MS	Training cohort: cancer patients *n* = 96	+	+	Shorter overall survival	Cer (d18:1_24:1), SM (d18:2_16:0), PC (16:0-16:0)	-
						Validation cohort: cancer patients *n* = 63					
	Garcia et al. 2018	Spain	Urinary EV	LC-MS	Database (LIPID MAPS)	Cancer patients *n* = 31 / Control *n* = 14	-	-	Advanced stage	-	Cer, PC (30:0)
Breast cancer	Silva et al. 2020	Portugal	Tissue	MALDI-TOF MS	Details unknown	Tumor *n* = 28 / Normal tissue *n* = 28	-	-	Advanced stage	Ratio of LPC (16:0) to PC (16:0_18:2)	-
	Hosokawa et al. 2017	Japan	Tissue	MALDI-IMS	Tandem MS	Tumor *n* = 9	-	-	Higner recurrence	PC (32:1)	-
	Tsuchida et al. 2016	Japan	Tissue	LC-MS	Radioisotope-labeled standard	Tumor *n* = 35	-	-	Higher lymph node metastasis	S1P	-
	Ikarashi et al. 2021	Japan	Serum	LC-MS	Radioisotope-labeled standard	Cancer patients *n* = 126	-	-	Advanced stage and T-factor Higher lymph node metastasis	S1P	-
	Buentzel et al. 2021	Germany	Serum EV	FIA-MS	Software (MetIDQ Carbon)	Cancer patients *n* = 78	-	-	Shorter overall survival	LPC (26:0), PC (38:5)	-
						Control = 30					
Lung cancer	Takanashi et al. 2020	Japan	Tissue	LC-MS	Tandem MS	Tumor *n* = 20	-	-	Higher recurrence	SM (d35:1)	-
	Takanashi et al. 2023	Japan	Tissue	LC-MS	Tandem MS	Tumor *n* = 26	-	-	Advanced T-facter Higner pleural invasion	PC	-
						Tumor *n* = 18			Higher recurrence	-	*m/z* 736.5276 (not identified)
	Takanashi et al. 2021	Japan	Tissue	LC-MS	Tandem MS	Tumor *n* = 11	-	-	Higher recurrence	-	SM (t34:1)
Ovarian cancer	Salminen et al. 2021	Finland	Serum	LC-MS	Software (Analyst)	Cancer patients *n* = 499 / Control *n* = 212	+	+	Shorter disease-free survival Shorter overall survival	Ratio of Cer (d18:1_18:0) to PC (O-38:4)	-
	Yang et al. 2020	China	Serum	LC-MS	Software (MassHunter Qualitative Analysis)	Ascities *n* = 249 / Control *n* = 188	-	-	Higher recurrence	LPC (P-15:0), PC	-
	Li et al. 2017	China	Serum	LC-MS	Software (MassHunter Qualitative Analysis)	Cancer patients *n* = 70	-	-	Higner recurrence	-	LPC (20:5), LPC, SM, PC
									Shorter recurrence-free period	-	TG
Bladder cancer	Sahu et al. 2017	USA	Tissue	GC-MS / LC-MS	Software (Metabolon)	Tumor *n* = 72 / Normal tissue *n* = 31	+	+	Higher muscle invasion	arachidonate, PG, thromboxane B2	-
	Piyarathna et al. 2017	USA	Tissue	LC-MS	Software (LipidBlast)	Tumor *n* = 126 / Normal tissue *n* = 39	-	-	Advanced stage	DG, PI	PC, PE, PS
Intrahepatic cholangiocarcinoma	Huizing et al. 2023	Netherlands	Tissue	MALDI-IMS	Tandem MS	Tumor *n* = 17	-	-	Shorter disease-free survival	Ratio of unsaturated to saturated ST species	-
						Normal tissue *n*=16					
	Li et al. 2022	China	Tissue	MALDI-IMS	Database (HMDB, LIPID MAPS)	Training cohort: tumor *n* = 5	+	-	Advanced stage	FA (22:4), GlcCer (d18:1/12:0), PA (P-18:0_0:0)	-
						Validation cohort: tumor *n* = 5				-	LPA (16:0), LPE (16:0), PE
Pancreatic cancer	Wolrab et al. 2022	Czech	Serum	UHPSFC-MS	Software (LipidQuant, ALEX, MassHunter Qualitative Analysis)	Training cohort: cancer patients *n* = 430 / control *n* = 268	+	+	Shorter overall survival	Cer (36:1), ratio of PC (32:0) to LPC (18:2)	-
						Validation cohort: cancer patients *n* = 116 / control *n* = 16					
	Tao et al. 2019	China	Serum EV	LC-MS	Software (MS-DIAL)	Training cohort: cancer patients *n* = 22 / control = 17	+	-	Shorter overall survival	PE (16:0_18:1)	-
						Validation cohort: cancer patients *n* = 48 / control = 40					
Renal cell carcinoma	Tamura et al. 2019	Japan	Tissue	DESI-IMS	Database (LIPID MAPS)	Tumor *n* = 47	-	-	Shorter progression-free survival	-	Oleic acid
	Manzi et al. 2023	Argentina	Serum	LC-MS	Software (Progenesis QJ)	Cancer patients *n* = 41	-	-	Higher recurrence	-	Ratio of PC to linoleic acid
Cervical cancer	Zhou et al. 2019	China	Serum	LC-MS	Software (Progenesis QJ)	Cancer patients *n* = 90	-	-	Higher recurrence Higher distant metastasis	PG (12:0_13:0)	LacCer (d18:1_16:0) PC (15:0_16:0)
Chordoma	Corte et al. 2019	Italy	Tissue	LC-MS	Software (Analyst)	Tumor *n* = 15	-	-	Higher Ki-67 expression	Cer, DHCer	-
Endometrial cancer	Delage et al. 2018	Canada	Serum	LC-MS	Software (Metabolon)	Cancer patients *n* = 36 / Control *n* = 18	-	-	Higher recurrence	Cer	-
Liver cancer	Lu et al. 2018	Singapore	Tissue	LC-MS	Database (METLIN, LIPID MAPS)	Training cohort: cancer patients *n* = 50 / control *n* = 24	+	+	Advanced stage	-	PE
			Serum			Validation cohort: cancer patients *n* = 18 / control *n* = 40					
Malignant mesothelioma	Chen et al. 2022	China	Serum	LC-MS	Software (LipidSearch)	Cancer patients *n* = 25	-	-	Shorter overall survival	Ratio of Hex1Cer (t18:1_24:1) to TG	-
						Control *n* = 32					
Nasopharyngeal carcinoma	Huang et al. 2022	China	Tissue	LC-MS	Details unknown	Tumor *n* = 100	-	-	Higher lymph node metastasis	DG (14:0_18:3), TG	PA (18:1_22:5)
									Higher distant metastasis	PE (16:0_20:3), PG (14:0_20:3), PS	DG, TG (40:0)
Oral cancer	Faedo et al. 2022	Brazil	Serum	LC-MS	Software (Analyst)	Cancer patients *n* = 56 / Control *n* = 58	-	-	Shorter overall survival	GlcCer (d18:1_24:1)	-
										-	Cer (d18:1_24:0) DHCer (d18:1_24:0) SM (d18:1_24:0)
			Tissue			Tumor *n* = 42 / Normal tissue *n* = 42			Shorter overall survival	Cer (d18:0_24:0), DHSM (d18:1_24:0)	-

*Molecules belonging to the same lipid class, if there are two or more of them, are collectively described as that lipid class

AcCa, acylcarnitine; CE, cholestelyl ester; Cer, ceramide; DESI-IMS, desorption electrospray ionization-imaging mass spectrometry; DG, diacylglycerol; DHCer, dihydroceramide; DHSM, dihydrosphingomyelin; FA, fatty acid; FAHFA, fatty acid ester of hydroxy fatty acid; FIA-MS, flow injection analysis-mass spectrometry; GC-MS, gas chromatogram-mass spectrometry; GlcCer, glucosylceramide; Hex1Cer, hexosylceramide; iEESI-MS, internal extractive electrospray ionization-mass spectrometry; LacCer, lactosylceramide; LC-MS, liquid chromatography–mass spectrometry; LPA, lysophosphatidic acid; LPC, lysophosphatidylcholine; LPE, lysophosphatidylethanolamine; LPI, lysophosphatidylinositol; LPS, lipopolysaccharide; *m/z*, mass to charge ratio; MALDI-IMS, matrix-assisted laser desorption ionization-imaging mass spectrometry; MALDI-TOF MS, matrix-assisted laser desorption ionization time-of-flight mass spectrometry; MS, mass spectrometry; PA, phosphatidic acid; PC, phosphatidylcholine; PE, phosphatidylethanolamine; PG, prostaglandin; PI, phosphatidylinositol; PS, phosphatidylserine; S1P, sphingosine-1-phosphate; SFA, saturated fatty acid; SM, sphingomyelin; ST, sulfatide; TG, triglyceride; UFA, unsaturated fatty acid; UHPSFC-MS, ultra-high performance supercritical fluid chromatography–mass spectrometry

#### Cancer types

**Fig. 4:**
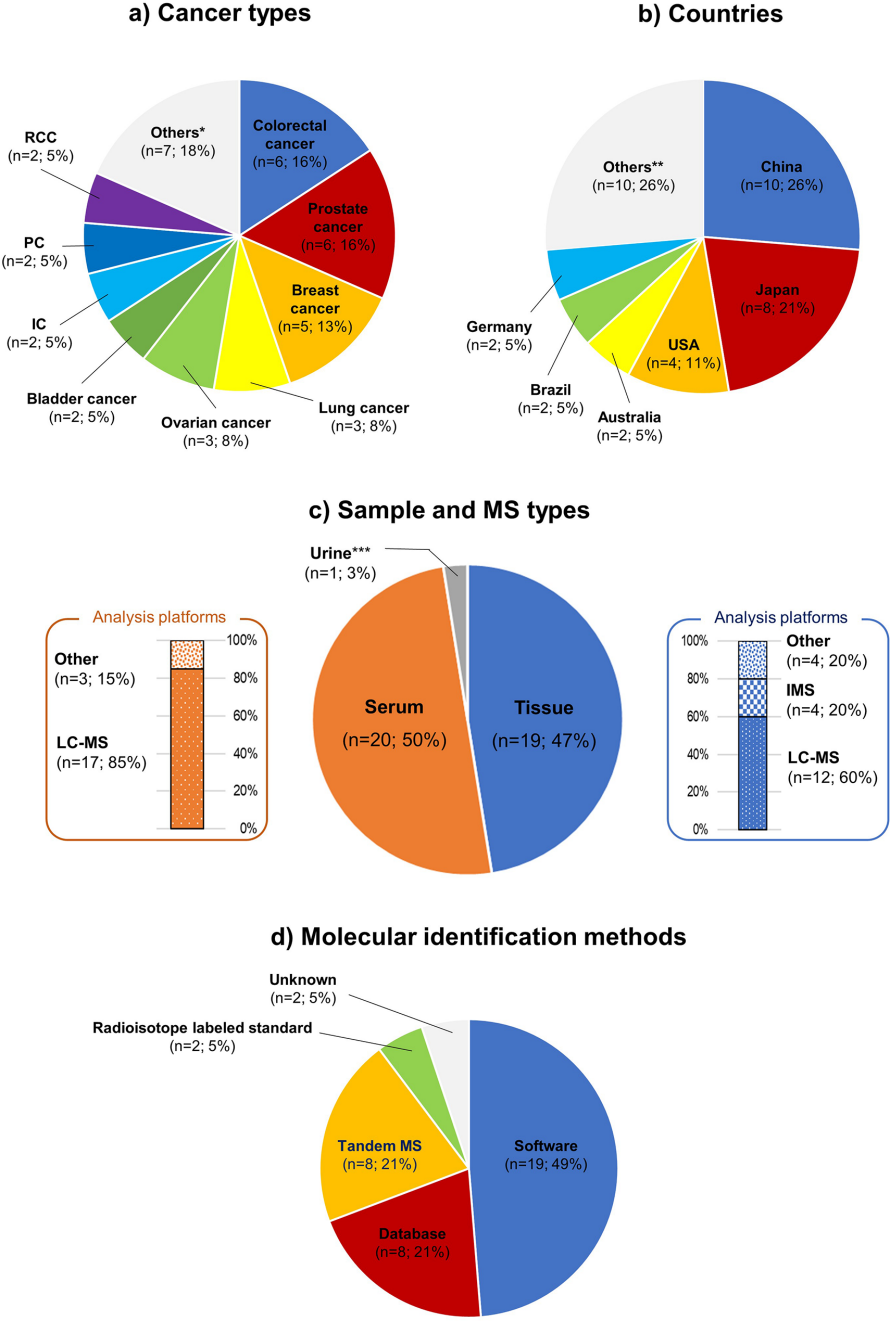
Diagrams showing the proportion of studied cancer types (**a**), countries (**b**), sample and MS types (**c**), and molecular identification methods (**d**) * Others included cervical cancer, chordoma, endometrial cancer, liver cancer, malignant mesothelioma, nasopharyngeal carcinoma, and oral cancer; each reported number was one (3%), respectively ** Others included Argentina, Canada, Czech, Finland, Italy, Netherlands, Portugal, Romania, Singapore, and Spain; each reported number was one (3%), respectively *** Analysis platform was LC-MS. IC, intrahepatic cholangiocarcinoma; IMS, imaging mass spectrometry; LC-MS, liquid chromatography - mass spectrometry, MS, mass spectrometry; PC, pancreatic cancer; RCC, renal cell carcinoma

The included studies were performed on 16 cancer types. The number of studies reported for each cancer type is as follows (Fig. 4a): there were six studies (16%) each on colorectal [24–29] and prostate cancer [30–35], five (13%) on breast cancer [36–40], three (8%) each on lung [41–43] and ovarian cancer [44–46], two (5%) on bladder cancer [47, 48], intrahepatic cholangiocarcinoma [49, 50], pancreatic cancer [51, 52], and renal cell carcinoma [53, 54], one (3%) each from cervical cancer [55], chordoma [56], endometrial cancer [57], liver cancer [58], malignant mesothelioma [59], nasopharyngeal carcinoma [60], and oral cancer [61].

#### Countries

The included studies were reported from 16 countries. The number of studies reported for each country is as follows (Fig. 4b): 10 studies (26%) were reported from China [24, 26, 28, 45, 46, 50, 52, 55, 59, 60], eight (21%) from Japan [31, 37–39, 41–43, 53], four (11%) from the USA [30, 33, 47, 48], two (5%) each from Australia [32, 34], Brazil [29, 61], and Germany [25, 40], one (3%) each from Argentina [54], Canada [57], Czech [51], Finland [44], Italy [56], Netherlands [49], Portugal [36], Romania [27], Singapore [58], and Spain [35].

#### Sample and MS types

The sample types used in the studies were tissue (19 cases; 47%) [24, 25, 30, 31, 36–38, 41–43, 47–50, 53, 56, 58, 60, 61], serum (20 cases; 50%) [26–29, 32–34, 39, 40, 44–46, 51, 52, 54, 55, 57–59, 61], and urine (one case; 3%) [35]. In two cases of serum and one urine, extracellular vesicle (EV) was used as the measurement materials [35, 40, 52]. For the analysis of tissue samples, the most commonly used analysis platform was liquid chromatography–tandem mass spectrometry (LC-MS), accounting for 12 cases (60%) [30, 31, 38, 41–43, 47, 48, 56, 58, 60, 61], followed by imaging mass spectrometry (IMS) [37, 49, 50, 53] and other methods [24, 25, 36, 47], each with four cases (20%). In the analysis of serum samples, LC-MS was predominantly used in 17 cases (85%) [26–28, 32–34, 39, 44–46, 52, 54, 55, 57–59, 61], with other methods being used in three cases (15%) [29, 40, 51]. LC-MS was also employed to analyze urinary EVs (Fig. 4c) [35].

#### Molecular identification methods

In the studies, the molecular identification methods used were predominantly software-based, accounting for 19 cases (49%) [25, 26, 28, 30, 33, 40, 44–48, 51, 52, 54–57, 59, 61], followed by databases in eight cases (21%) [24, 27, 29, 33, 35, 50, 53, 58]. Direct verification of raw-tandem MS (MS/MS) data and the use of radioisotope-labeled standards were each employed in eight (21%) [31, 32, 34, 37, 41–43, 49] and two cases (5%) [38, 39]. There were also two cases (5%) [36, 60] where the details were not specified in the text. Within the software category, Analyst was the most frequently used in five cases (24%) [26, 30, 44, 56, 61], followed by Mass Hunter Qualitative Analysis in three cases (14%) [45, 46, 51]. ALEX [25, 51], Metabolon [47, 57], MS-DIAL [28, 52], Progenesis QI [54, 55] and LipidSearch [33, 59] were used each in two cases (10%). LipidBlast [48], LipidQuant [51], and MetIDQ Carbon [40] were each used each in one case (5%). In terms of databases, LIPID MAPS (https://www.lipidmaps.org/) was the most common, utilized in six cases (50%) [27, 29, 35, 50, 53, 58], followed by HMDB (https://hmdb.ca/) in three cases (25%) [27, 33, 50], METLIN (https://metlin.scripps.edu/landing_page.php?pgcontent=mainPage) in two cases (17%) [33, 58], and NIST Mass Spectral Library (https://chemdata.nist.gov/) in one case (8%) [24] (Fig. 4d).

#### Study size and design

**Fig. 5:**
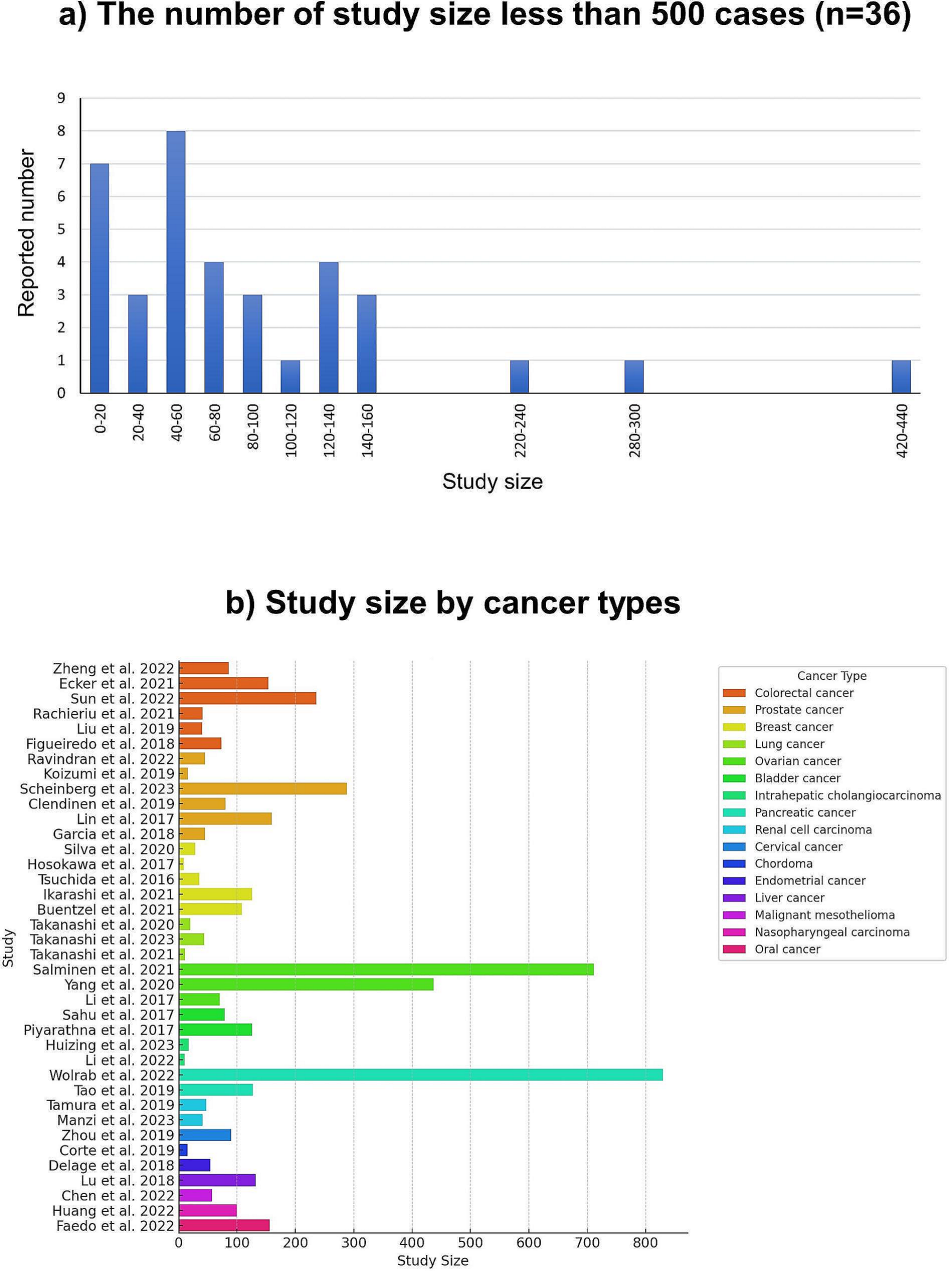
(**a**) Histogram of study size. 33 studies included fewer than 160 cases, demonstrating a bias in the distribution. (**b**) Study size by cancer types. The cancer types with studies involving more than 100 cases were colorectal cancer, prostate cancer, breast cancer, ovarian cancer, bladder cancer, pancreatic cancer, liver cancer, nasopharyngeal carcinoma, and oral cancer

The median number of cases analyzed was 72 (range: 9–830). In the histogram of case numbers (Fig. 5a), 33 studies included fewer than 160 cases, demonstrating a bias in the distribution of case numbers. In comparison, only five studies exceeded 160 cases [26, 32, 44, 45, 51]. Regarding the breakdown by cancer type, the cancer types with studies involving more than 100 cases were colorectal cancer, prostate cancer, breast cancer, ovarian cancer, bladder cancer, pancreatic cancer, liver cancer, nasopharyngeal carcinoma, and oral cancer. Among these, studies of a significantly larger scale exceeding 500 cases were conducted for ovarian cancer (*n* = 711) [44] and pancreatic cancer (*n* = 830) [51] (Fig. 5b). Studies that performed validation analysis using independent cohorts accounted for nine studies (24%) [25, 32, 34, 44, 47, 50–52, 58], of which seven studies (18%) [25, 32, 34, 44, 47, 51, 58] used multi-center cohorts, with a median number of cases being 159 (range: 79–830).

#### Evaluated prognostic outcomes (prognosis or prognostic factors)

**Fig. 6:**
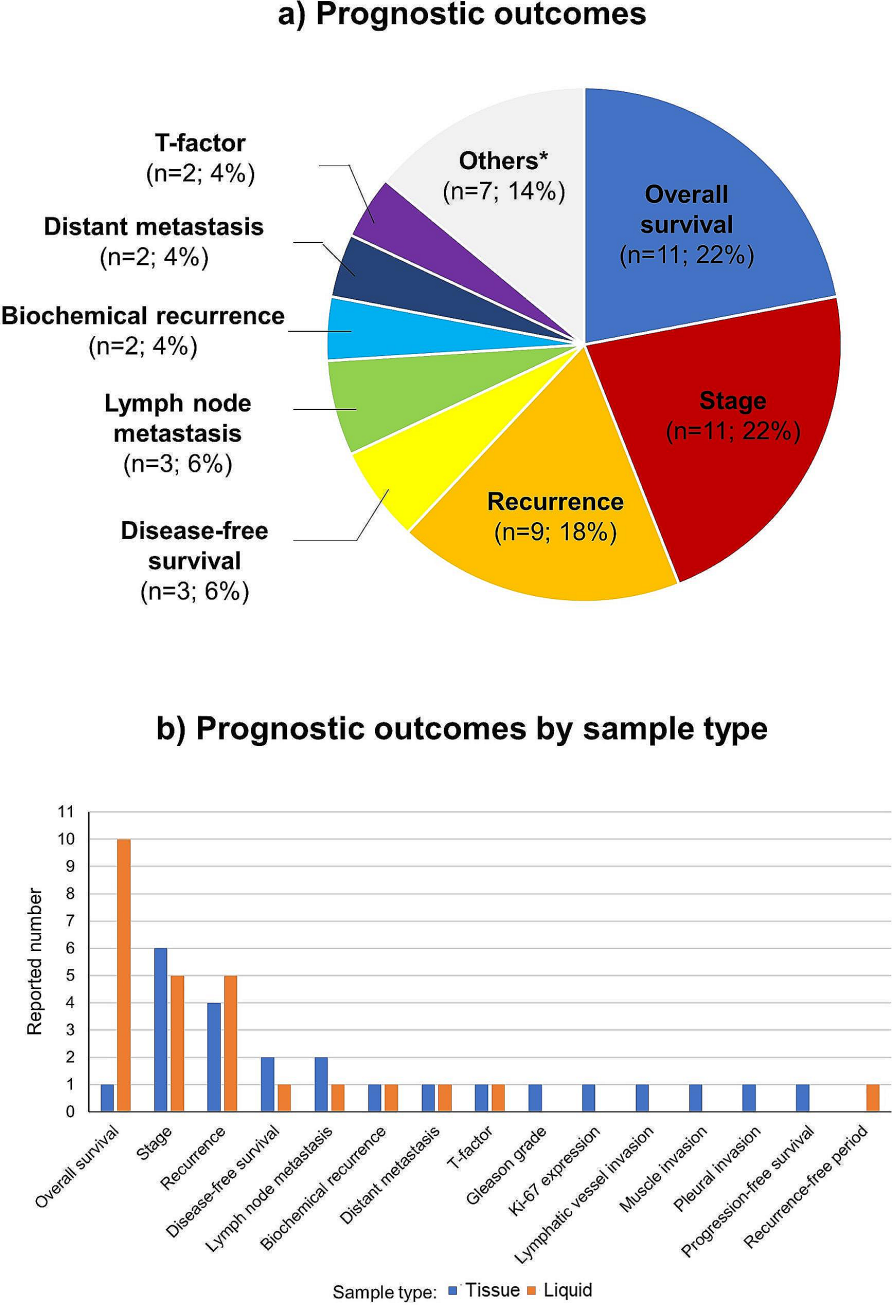
(**a**) Diagram showing the proportion of evaluated prognostic outcomes. (**b**) Evaluated outcome by sample type * Others included Gleason grade, Ki-67 expression, lymphatic vessel invasion, muscle invasion, pleural invasion, progression-free survival, and recurrence-free period; each reported number was one (2%), respectively

The evaluated prognostic outcomes were a total of 15 types (Fig. 6). Among them, overall survival [26, 29, 32, 34, 40, 44, 51, 52, 59, 61] and stage [24, 27, 28, 31, 35, 36, 39, 48, 50, 58] were the most frequent, with 11 cases each (22%), followed by recurrence with nine cases (18%) [37, 41–43, 45, 46, 54, 55, 57], and disease-free survival [25, 44, 49] and lymph node metastasis [38, 39, 60] with three cases each (6%). Evaluation items with two or fewer reports were characterized by cancer type-specific assessments, including biochemical recurrence (for prostate cancer) [30, 33], distant metastasis [55, 60], T-factor [39, 42], each with two cases (4%), Gleason grade (for prostate cancer) [30], Ki-67 expression (for chordoma) [56], lymphatic vessel invasion [25], muscle invasion (for bladder cancer) [47], pleural invasion (for lung cancer) [42], progression-free survival [53], and recurrence-free period [46], each with one case (2%) (In cases where a certain outcome within the same study was assessed using different sample types, it was counted independently.). When we examine the breakdown of each evaluated outcome by sample type (tissue, liquid samples [including serum, serum EV, and urinary EV]), overall survival, which was the most frequent, was predominantly based on liquid samples (tissue: one case, liquid samples: 10 cases), whereas for stage (tissue: six cases, liquid samples: five cases) and recurrence (tissue: four cases, liquid samples: five cases), the sample type distribution was approximately equal. Gleason grade, Ki-67 expression, lymphatic vessel invasion, muscle invasion, and plural invasion, all related to histopathological examinations, were exclusively assessed using tissue samples.

#### Observed lipid biomarkers

**Fig. 7:**
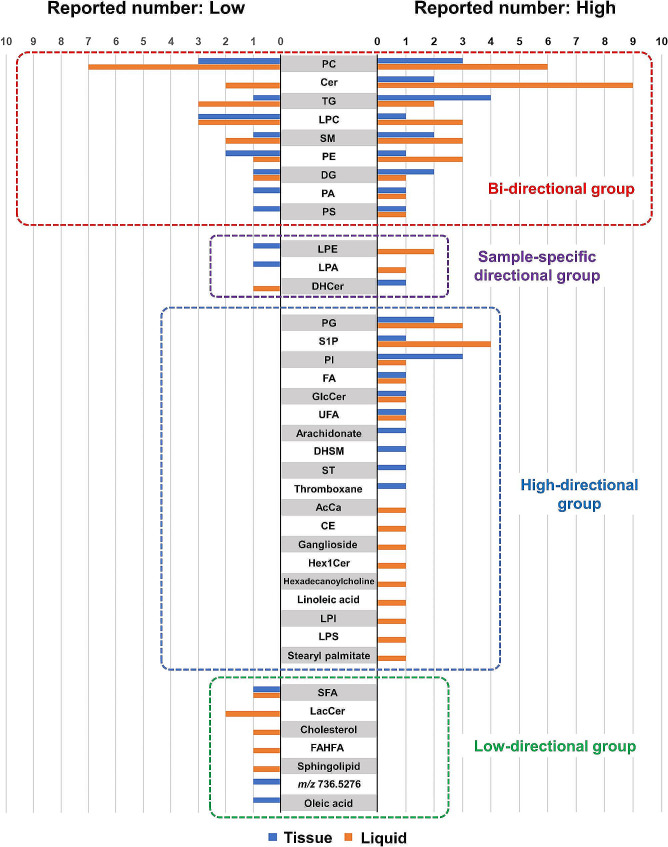
Butterfly chart of the observed lipid biomarkers. The blue bars represent tissue samples, while the orange bars indicate liquid samples. The right half of the chart shows the number of reports where lipid markers are high, and the left half indicates the number of reports where they are low. In both directions, the outcomes suggest a tendency towards poor prognosis AcCa, acylcarnitine; CE, cholestelyl ester; Cer, ceramide; DG, diacylglycerol; DHCer, dihydroceramide; DHSM, dihydrosphingomyelin; FA, fatty acid; FAHFA, fatty acid ester of hydroxy fatty acid; GlcCer, glucosylceramide; Hex1Cer, hexosylceramide; LacCer, lactosylceramide; LPA, lysophosphatidic acid; LPC, lysophosphatidylcholine; LPE, lysophosphatidylethanolamine; LPI, lysophosphatidylinositol; LPS, lipopolysaccharide; PA, phosphatidic acid; PC, phosphatidylcholine; PE, phosphatidylethanolamine; PG, prostaglandin; PI, phosphatidylinositol; PS, phosphatidylserine; S1P, sphingosine-1-phosphate; SFA, saturated fatty acid; SM, sphingomyelin; ST, sulfatide; TG, triglyceride; UFA, unsaturated fatty acid

We present the number of reports on lipid markers found in tissue or liquid samples associated with poor prognosis using a butterfly chart (Fig. 7). In cases where lipid species (same head group but different molecular species within the same study) exhibited low and high values, they were counted independently. Also, in cases where different prognostic outcomes were reported for the same molecule, they were counted independently.

A total of 38 different lipid markers were reported. Of these, nine types (24%) were observed in both high and low values in either tissue or liquid samples, forming the “bi-directional group”, implying a poor prognosis. The “sample-specific directional group”, which shows different trends in lipid marker levels depending on the sample types, consisted of three types (8%). There were 19 types (50%) in the “high-directional group”, which only showed elevated levels, and seven types (18%) in the “low-directional group”, which only exhibited reduced levels.

Among the lipid markers categorized in the bi-directional group, PC, a principal constituent of the cell membrane, had the highest number of reports at 19 [27, 30, 33–37, 40, 42, 44–46, 48, 51, 54, 55], followed by ceramide (Cer) with 13 reports [26, 27, 32–35, 44, 51, 56, 57, 61], TG [25, 28, 30, 32, 33, 46, 59, 60] and lysophosphatidylcholine (LPC) [24, 30, 33, 36, 40, 45, 46, 51] with 10, sphingomyelin (SM) with eight [27, 30, 33, 34, 41, 43, 46, 61], PE with seven [27, 33, 48, 50, 52, 58, 60], diacylglycerol (DG) with five [29, 33, 48, 60], and both phosphatidic acid (PA) [27, 50, 60] and phosphatidylserine (PS) [26, 48, 60] with three each. In the sample-specific directional group, lysophosphatidylethanolamine (LPE) had three reports [26, 33, 50], and both lysophosphatidic acid (LPA) [33, 50] and dihydroceramide (DHCer) [56, 61] had two each. In the high-directional group, PG [33, 47, 55, 60] and S1P [33, 38, 39] had the highest number of reports at five, followed by PI with three [31, 33, 48], and FA [27, 50], glucosylceramide (GlcCer) [50, 61], and unsaturated fatty acid (UFA) [32, 49] each with two. The remaining 13 types of lipid markers were reported once each. In the low-directional group, saturated fatty acid (SFA) [32, 49] and lactosylceramide (LacCer) [55] had two reports each, with the remaining five types of lipid markers reported once each.

## Discussion

The trend in the number of research reports applying lipidomics in cancer for prognosis prediction has been increasing since 2016, and it was surmised that this field of research has been active for less than a decade. In addition to the widespread use of conventional LC-MS in lipidomics, the increasing popularity of a relatively new MS method, IMS [62], and the emerging limitations in prognostic prediction in pathological diagnosis, namely the lack of reproducibility due to subjective judgments [17–19], were considered to be factors behind the increase in research reports.

Our systematic search identified 38 publications that matched the inclusion criteria. In the quality assessment using QUADAS-2, the majority of the studies adopted a retrospective design, and due to the exploratory nature of the studies, it was impossible to pre-specify the threshold of the index test, resulting in 76.3% and 92.1% of reports being judged as “high risk” in patient selection and index test, respectively. Regarding study size and design, analyses based on small sample sizes were standard (median: 72 cases). Furthermore, only nine studies (24%) utilized an independent validation cohort within the same research. Therefore, the evidence level of the reports included in this review was considered low, suggesting that this research field is still not well-established and is immature. Future research should focus on validation through prospective studies using large cohorts based on these exploratory analysis results to accumulate high-level evidence.

There were 16 cancer types in which lipidomic research has been conducted, ranging from common to rare types. The top four reported cancer types (colorectal, prostate, breast, lung cancers) corresponded to the most commonly diagnosed cancer types in global cancer statistics in 2020 (breast (11.7%), lung (11.4%), colorectal (10.0%) and prostate (7.3%) cancers) [63]. It is speculated that cancers with a higher patient population also have a greater demand for research and supply of specimens.

The top three countries with the highest number of research reports were China, Japan, and the USA. These three nations correspond to the top three countries with abundant funding for cancer research from 2016 to 2020 [64]; this funding availability might facilitate access to expensive equipment, such as mass spectrometers, creating an environment conducive to advanced cancer research.

The sample types used for prognostic prediction were liquid samples (serum and urine) and tissue samples, which were almost equivalent in their application. Liquid samples have the advantage of being less invasive during collection compared to tissue samples. They also excel in continuous monitoring even after surgical removal of tumors, suggesting their potential application as recurrence markers for postoperative monitoring. In clinical implementation, utilizing enzyme-linked immunosorbent assay (ELISA) for lipid measurement can be possible option [65]. On the other hand, tissue samples can only be obtained through invasive procedures such as surgery or biopsy. They have the advantage of directly analyzing the biological characteristics of tumor tissues (such as malignancy, directly linked to prognosis) compared to serum or urine samples. Immunostaining of enzymes involved in the synthesis/degradation of lipid markers identified by MS may enable clinical implementation of prognostic markers. Studies analyzing paired tissue and serum samples for the same lipid markers were limited to the analysis of liver cancer by Lu et al.: both sample types showed low PE species levels in advanced-stage patients. The advantage of using paired samples is that it provides more options for clinical implementation of prognostic markers, such as ELISA in serum and immunostaining in tissue, depending on the identified lipid markers.

The most commonly used analysis platform across liquid and tissue samples was LC-MS, a widely used method in lipidomics [62]. In studies using tissue samples, IMS was employed in 20% (*n* = 4) of cases. LC-MS analysis requires homogenization of tissue and lipid extraction via the Bligh & Dyer method [41], frequently needing a relatively large sample volume. Conversely, IMS allows direct measurement of molecules in thin tissue sections and retains spatial information of measured molecules, enabling investigation of the molecular distribution corresponding to biological structures. With advancements in MS, the application of IMS in lipidomics is increasing [62]. Three of the IMS studies in this review used matrix-assisted laser desorption ionization (MALDI)-IMS, and one used desorption electrospray ionization (DESI)-IMS. MALDI-IMS, the most prevalent IMS method, allows analysis of a broad mass range of molecules by ionizing them with various matrices applied to the sample. Using this method, Hosokawa et al. discovered that PC (32:1) was specifically distributed in tumor regions in recurrent cases of triple-negative breast cancer patients [37]. Similarly, Huizing and Li et al. analyzed intrahepatic cholangiocarcinoma, finding correlations between high ratios of unsaturated to saturated sulfatide (ST) species and shorter disease-free survival, and high levels of FA (22:4), PA (P-18:0_0:0), GlcCer (d18:1/12:0) but low levels of LPA (16:0), LPE (16:0), and PE species with advanced stages [49]. In these studies, IMS effectively visualized tumor-specific molecular distributions by analyzing tumor and normal parts of tissue samples. DESI-IMS, which ionizes molecules without pre-treatment like matrix application, avoids signal interference from the matrix, allowing visualization of free FA and lipid mediators, which are challenging to measure with MALDI-IMS [62]. Tamura et al. utilized this method to reveal that low level of oreic acid distributed in clear cell renal cell carcinoma tumor regions correlated with shorter progression-free survival [53]. The application of IMS in this research field is anticipated to expand.

In lipidomics, several methods exist for identifying lipid molecules. Firstly, a method involves comparing the observed mass-to-charge ratio (*m/z*) value of the focused molecule with known compounds’ *m/z* reported in various databases, typically allowing a mass error range within 1–20 ppm; this approach is advantageous due to its simplicity and low cost. In cases where the assignment is unambiguous, MS/MS data analysis is employed to identify characteristic fragment ion patterns specific to individual lipid molecules; this method is more reliable but requires expertise and is labor-intensive [62]. Recent developments have seen the creation of software capable of identifying multiple lipid molecules based on MS/MS data, a powerful tool for exploratory non-target lipidomics, albeit with a certain probability of identification errors [66]. For targeted analysis in case the focused molecules are pre-specified, methods involving the measurement of radioisotope-labeled standards alongside samples are used [38, 39]. This review found that software-based molecular identification methods comprise 49% of the methods employed, making it the most popular approach. The predominance of software usage may be due to most studies being exploratory non-target analyses. While most lipid molecule identification software lacks published data on identification accuracy, MS-DIAL has demonstrated a low false discovery rate of 1.50–2.08% [66]. Direct identification from MS/MS data is advisable for a few targeted molecules, whereas software is practical for identifying multiple molecules.

Regarding study size and design, the median number of cases used was 72, indicating that most studies employed small cohorts, often limited to 160 cases or fewer. One reason may be the need to stock raw samples for lipid analysis using MS, limiting the number of available specimens. Another consideration is the difficulty in analyzing a large number of cases. In non-targeted lipidomics, the lipid species analyzed can number thousands per case [41, 43], data analysis becoming a bottleneck as the number of cases increases. This trend is particularly pronounced in analyses using IMS due to the addition of spatial information of molecules, prompting attempts to develop methods for simplifying the analysis of extensive distribution data [67]. Studies using validation cohorts were limited to nine (24%). As previously mentioned, the difficulty in securing sufficient samples for preparing validation cohorts is a likely reason for this limitation. The study with the smallest number of cases was MALDI-IMS analysis of intrahepatic cholangiocarcinoma (training cohort: *n* = 5, validation cohort: *n* = 5) [50]. Given the noted bottleneck in IMS analysis, it may be practical for studies using IMS to split small cohorts for validation purposes.

The most frequent prognostic outcome evaluated was overall survival (11 cases), of which serum samples were used in 10 cases. Given that the ultimate goal of cancer patient treatment strategies is the improvement of overall survival, it is natural that this outcome was the most commonly used evaluation criterion. The predominant use of serum samples in most cases can be attributed to several advantages: they enable stratification in treatment choices through prognostic predictions even for patients who are not scheduled for surgery (and thus cannot provide tissue samples), and they allow for post-treatment ongoing prognostic evaluation due to their relatively non-invasive nature and the feasibility of repeated sampling. The second most common evaluated outcome was the stage of cancer. Conventional pathological diagnoses have been problematic due to subjective judgments and lack of reproducibility [17–19]. Therefore, they might support the pathological stage diagnosis if the identified lipid molecules can be applied as novel biomarkers with high objectivity and reproducibility. Regarding the third most common factor, recurrence, if it becomes possible to select patient groups with a high risk of recurrence accurately, this would enable precise determination of post-surgical treatment strategies (such as the applicability of adjuvant chemotherapy), potentially improving patient prognosis.

In this review, we identified a total of 38 reported lipid markers. Due to the extensive range of lipid types reported and the space constraints of this review, we focused on discussing the main findings.

Most lipids constituting the membrane structure of mammalian cells are composed of five types, namely PC, PE, PS, SM and PI, with the rest accounting for only a few percent [68]. In this review, all five major lipids constituting the membrane structure were reported as prognostic markers, and they were frequently mentioned. Due to the predominant non-biased exploratory analyses in the included studies, lipids that are abundant in quantity were more likely to be identified as prognostic markers. Of these, PC, PE, PS, and SM were categorized in the bi-directional group. PC is the most abundant lipid species in the cell membrane structure, followed by PE. This hierarchy is consistent across different types of membrane structures, such as the plasma membrane and various organelles [68]. In cancer cells, the de-novo synthesis and uptake of PC and PE from the bloodstream or adipose tissue are enhanced in various cancer types to support their high proliferative capacity [5–8]. PS is externalized to the outer leaflet of the plasma membrane in cancer cells by phospholipid scramblases. Externalized PS on the surface of cancer cells supports immune evasion by controlling the infiltration of immune cells into tumors, thus enhancing cancer cell survival, drug resistance, and metastasis [69]. SM, constituting lipid rafts [2], is predominantly localized in the plasma membrane [68]. Lipid rafts hold and localize various signalling proteins, thereby enhancing the efficiency of signalling pathways that promote cancer cell proliferation. Thus, SM plays a crucial role in cancer cell proliferation and survival [2]. Next, we discuss less abundant lipids. Cer, the second most frequently reported lipid after PC, is a precursor of SM. In cancer cells, the accumulation of Cer can contribute to apoptosis and tumorigenesis [70]. TG, forming lipid droplets along with cholesterol in many aggressive cancer types with a “lipid accumulating phenotype”, provides an energy source under hypoxic stress [3]. TG blood level is considered a risk factor for lung cancer [71]. The bi-directional nature of lipid markers, showing both high and low values, may suggest that the balance between lipid supply and consumption varies among cancer types.

LPE, LPA, and DHCer were categorized in the sample-specific directional group. LPE and LPA evoke intercellular signal transduction through G-protein coupled receptors, exhibiting growth factor-like effects [3, 26]. In this review, low values in tissues and high values in liquid samples indicated a poor prognosis trend, although the mechanisms remain unclear. The synthesis of DHCer in cancer cells contributes to survival and treatment resistance through cytoprotective autophagy [72], and its high tissue levels are consistent with poor prognosis. However, the reason why low levels in liquid samples indicate poor prognosis is not clear [61]. If the lipid markers in this group consistently show high or low levels specific to the sample, it might be easy to apply them universally across different cancer types.

Lipids categorized in the high-directional group, PG, S1P, and PI, are representative signalling mediators. They contribute to cancer cell proliferation by activating the RAS, STAT3, and PI3K/AKT pathways, respectively [9–11]. Regarding UFA, Scheinberg et al. focused on Cer species with the same length of fatty acid side chains in serum samples of prostate cancer patients. They adopted a high ratio of Cer containing UFA (C24:1) to Cer containing SFA (C24:0) as a poor prognostic factor in the overall survival prediction model [32]. Huizing et al. demonstrated that a high ratio of unsaturated to saturated ST species in intrahepatic cholangiocarcinoma tissue correlates with shorter disease-free survival [49]. In cancer cells, de novo synthesis of FAs is upregulated. The synthesized SFAs are converted to UFAs by enzymes such as stearoyl-CoA desaturases. Since SFAs are more prone to lipid peroxidation, leading to ferroptotic cell death, an increase in the relative amount of UFAs to SFAs in high-grade malignant tissues, due to enhanced expression of stearoyl-CoA desaturases, enhances the oxidative stress resistance of cancer cells [3]. Therefore, an increase in the relative amount of UFAs is considered a poor prognostic factor. Consequently, a decrease in the relative amount of SFAs is counted as a poor prognostic factor in the low-directional group in Fig. 7. For other lipid species, many are reported only in tissues or liquid samples, and the number of reports is limited, suggesting the potential for further research on unexplored sample and cancer types. If the lipid markers in this group consistently show high values irrespective of the sample, they might be universally applicable across different samples and cancer types. However, their categorization might change for lipid markers with few reports as more studies are published.

**Fig. 8:**
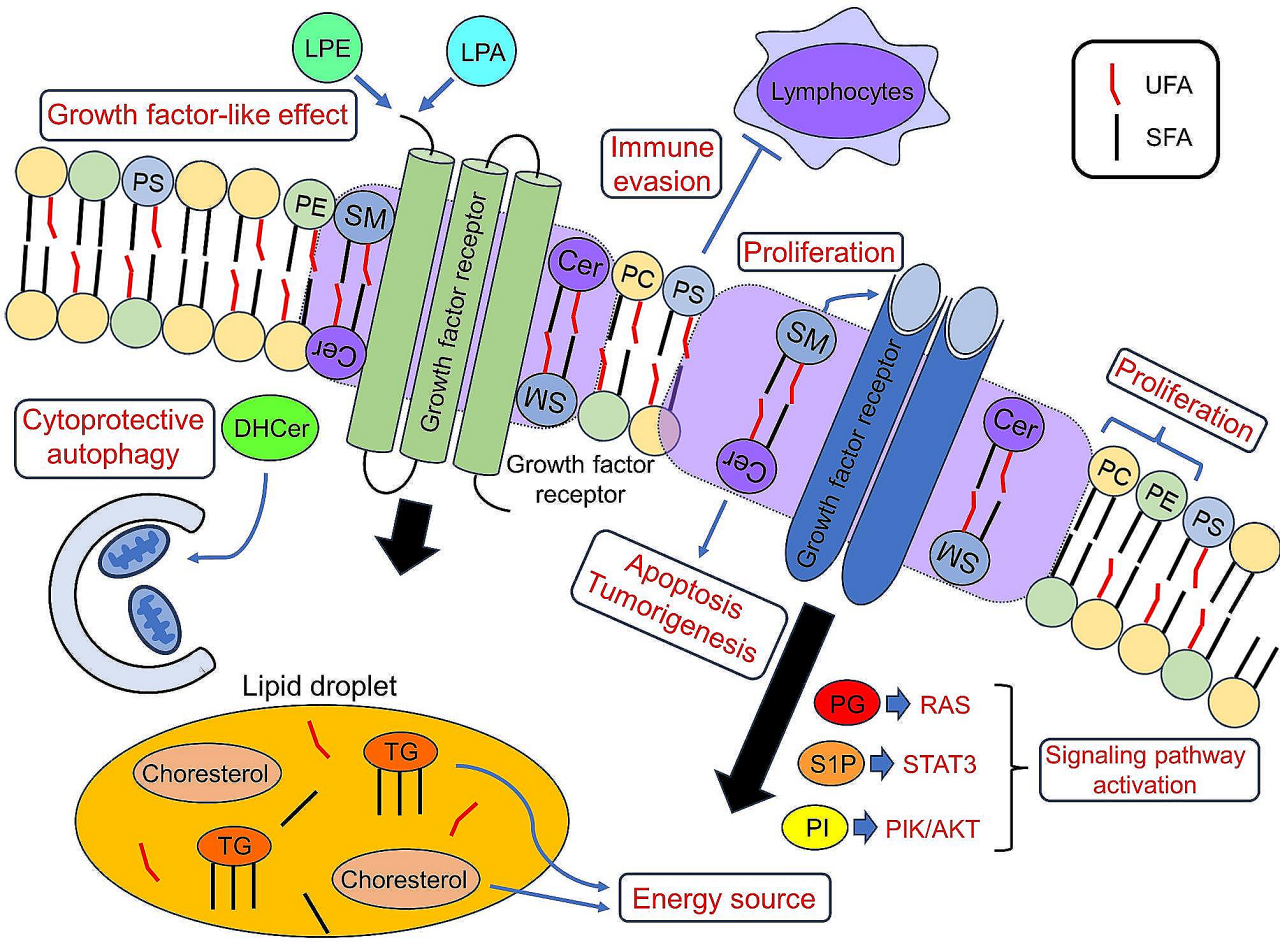
Prognostic lipid roles in cancer cells. Enhanced de-novo synthesis and uptake of PC and PE support proliferative capacity. PS on the surface of cancer cells supports immune evasion by controlling the infiltration of immune cells. SM constitutes lipid rafts in the plasma membrane and supports enhancing the efficiency of signalling pathways that promote cancer cell proliferation. Cer, a precursor of SM, contributes to apoptosis and tumorigenesis. TG, cholesterol, and FA, which form lipid droplets, provide an energy source. LPE and LPA evoke intercellular signal transduction through G-protein coupled receptors, exhibiting growth factor-like effects. DHCer contributes to survival and treatment resistance through cytoprotective autophagy. PG, S1P, and PI are signalling mediators contributing to cancer cell proliferation by activating the RAS, STAT3, and PI3K/AKT pathways. The relative amount of UFAs to SFAs enhances the oxidative stress resistance of cancer cells Cer, ceramide; DHCer, dihydroceramide; FA, fatty acid; LPA, lysophosphatidic acid; LPE, lysophosphatidylethanolamine; PC, phosphatidylcholine; PE, phosphatidylethanolamine; PG, prostaglandin; PI, phosphatidylinositol; PI3K/AKT: phosphoinositide 3-kinase/protein kinase B, PS: phosphatidylserine, S1P: sphingosine-1-phosphate, SFA: saturated fatty acid, SM: sphingomyelin, STAT3: signal transducer and activator of transcription 3, TG: triglyceride, UFA: unsaturated fatty acid

Finally, we will mention a few lipid markers categorized into the low-directional group. Both cholesterol and oleic acid (a type of FA) form lipid droplets within cells and are important energy sources for cancer cells [3]. Tamura et al. hypothesized that with the progression of cancer and increased energy consumption, the depletion of lipid storage beyond supply leads to a decrease in lipids serving as energy sources, which may be a poor prognostic factor [53]. We present a schema summarizing the prognostic lipid roles discussed above (Fig. 8).

## Conclusions

Our systematic review summarized 38 studies from the past decade that attempted prognostic prediction of cancer patients through lipidomics. The lipid markers reported were diverse, encompassing a total of 38 types. Among these, lipids that constitute the cellular membrane structure (such as PC, PE, PS, SM, and PI) have been identified as potential prognostic markers across various types of cancer. Future research is anticipated on the clinical application of these potential lipid markers.

### Electronic supplementary material

Below is the link to the electronic supplementary material.

Supplementary Material 1

## Data Availability

No datasets were generated or analysed during the current study.

## Abbreviations

Cer: ceramide
DESI: desorption electrospray ionization
DG: diacylglycerol
DHCer: dihydroceramide
ELISA: enzyme-linked immunosorbent assay
EV: extracellular vesicle
FA: fatty acid
GlcCer: glucosylceramide
IMS: imaging mass spectrometry
LacCer: lactosylceramide
LC-MS: liquid chromatography–tandem mass spectrometry
LPA: lysophosphatidic acid
LPC: lysophosphatidylcholine
LPE: lysophosphatidylethanolamine
MALDI: matrix-assisted laser desorption ionization
MS/MS: tandem mass spectrometry
PA: phosphatidic acid
PC: phosphatidylcholine
PE: phosphatidylethanolamine
PG: prostaglandin
PI: phosphatidylinositol
PI3K/AKT: phosphoinositide 3-kinase/protein kinase B
PS: phosphatidylserine
S1P: sphingosine-1-phosphate
SFA: saturated fatty acid
SM: sphingomyelin
ST: sulfatide
STAT3: signal transducer and activator of transcription 3
TG: triglyceride
UFA: unsaturated fatty acid
